# Gsmtx4 Alleviated Osteoarthritis through Piezo1/Calcineurin/NFAT1 Signaling Axis under Excessive Mechanical Strain

**DOI:** 10.3390/ijms24044022

**Published:** 2023-02-16

**Authors:** Xunshan Ren, Huangming Zhuang, Bin Li, Fuze Jiang, Yuelong Zhang, Panghu Zhou

**Affiliations:** 1 Department of Orthopedics, Renmin Hospital of Wuhan University, Wuhan 430072, China; 2 Division of Joint Surgery and Sports Medicine, Department of Orthopedic Surgery, Zhongnan Hospital of Wuhan University, Wuhan 430072, China

**Keywords:** piezo1, osteoarthritis, mechanical strain, chondrocyte, Gsmtx4

## Abstract

Excessive mechanical strain is the prominent risk factor for osteoarthritis (OA), causing cartilage destruction and degeneration. However, the underlying molecular mechanism contributing to mechanical signaling transduction remains unclear in OA. Piezo type mechanosensitive ion channel component 1 (Piezo1) is a calcium-permeable mechanosensitive ion channel and provides mechanosensitivity to cells, but its role in OA development has not been determined. Herein, we found up-regulated expression of Piezo1 in OA cartilage, and that its activation contributes to chondrocyte apoptosis. The knockdown of Piezo1 could protect chondrocytes from apoptosis and maintain the catabolic and anabolic balance under mechanical strain. In vivo, Gsmtx4, a Piezo1 inhibitor, markedly ameliorated the progression of OA, inhibited the chondrocyte apoptosis, and accelerated the production of the cartilage matrix. Mechanistically, we observed the elevated activity of calcineurin (CaN) and the nuclear transfection of nuclear factor of activated T cells 1 (NFAT1) under mechanical strain in chondrocytes. Inhibitors of CaN or NFAT1 rescued the pathologic changes induced by mechanical strain in chondrocytes. Overall, our findings revealed that Piezo1 was the essential molecule response to mechanical signals and regulated apoptosis and cartilage matrix metabolism via the CaN/NFAT1 signaling axis in chondrocytes, and that Gsmtx4 could be an attractive therapeutic drug for OA treatment.

## 1. Introduction

Osteoarthritis (OA) is the most common chronic joint disease, characterized by degeneration of articular cartilage and affecting more than 300 million people worldwide [1]. The risk factor of OA is complex: age, obesity, and metabolic and genetic factors have been proven to be involved in the development of OA [2]. Due to the unclear pathogenesis and the lack of effective treatment options, OA tends to progress continuously and impose a substantial economic burden. Identifying the pathogenesis and therapeutic target for OA is exceptionally urgent.

Excessive mechanical strain is the only obligatory factor in the development of OA, including stretch, shear, and strain [3]. Under pathologic conditions such as obesity, trauma, and joint instability, chondrocytes respond to excessive mechanical strain and manifest as apoptosis and inflammatory reaction, which accelerate the progression of OA [4]. Mechanosensitive ion channels are membrane proteins that translate mechanical stimuli into electrical or chemical signals and lead to physiological responses [5]. Piezo type mechanosensitive ion channel component 1 (Piezo1) is the first mechanical-sensitive ion channel reported on the surface of mammalian cells and contributes to numerous physiological and pathophysiological processes such as cell proliferation, senescence, differentiation, and death [6,7,8]. Piezo1 functions in growth plate chondrocytes and contributes to chondrogenesis and endochondral ossification during development [9]. In addition, Piezo1 responded to the abnormal mechanical stimulus, causing the apoptosis and senescence of chondrocytes [10,11]. However, the potential role and mechanisms of Piezo1 on the excessive mechanical strain-induced OA remain unclear.

Calcium is an essential second messenger in eukaryotic cells, regulating various physiological and biochemical processes, such as anabolic metabolism, phenotypic maintenance, and cell apoptosis [12,13]. High calcium content has been observed in OA cartilage and caused cartilage matrix degradation [14]. The calcineurin (CaN)/nuclear factor of the activated T cells (NFAT) signaling axis is the crucial signal downstream of calcium. CaN, a phosphatase sensitive to calcium concentration, can dephosphorylate the hyper-phosphorylated NFAT factors and causes their nuclear translocation [15]. Activated NFAT1 contributed to the expression of matrix metalloenzyme and inflammatory cytokines, which triggers OA [16]. Given that Piezo1 relays calcium in response to mechanical strain, we speculate that mechanical strain aggravated OA through the Piezo1/CaN/NFAT1 signaling axis.

Gsmtx4, a peptide isolated from the venom of a tarantula, could inhibit the activation of Piezo1. A series of evidence has abounded for the beneficial applications of Gsmtx4 in treating multiple diseases such as myocardial infarction, hereditary spherocytosis, and hypertension [17,18,19]. Therefore, targeting the Piezo1/CaN/NFAT1 signaling axis by Gsmtx4 has promising therapeutic potential for OA.

In this study, we found that the expression of Piezo1 was increased in the articular cartilage of OA patients and anterior cruciate ligament transection (ACLT)-induced OA rats. Activation or knockdown of Piezo1 could regulate chondrocyte apoptosis and disrupt the anabolic and catabolic homeostasis. Subsequently, we identified the CaN/NFAT1 signaling axis as the downstream signaling for Piezo1 modulation of chondrocyte apoptosis and the anabolic and catabolic homeostasis. Furthermore, we determined the therapeutic effects of the Piezo1 inhibitor Gsmtx4 in OA rats. Our results revealed the novel mechanism of OA and provided a new therapeutic target for OA.

## 2. Results

### 2.1. Piezo1 Was Up-Regulated in the Articular Cartilage of OA Patients and Rats

**Figure 1:**
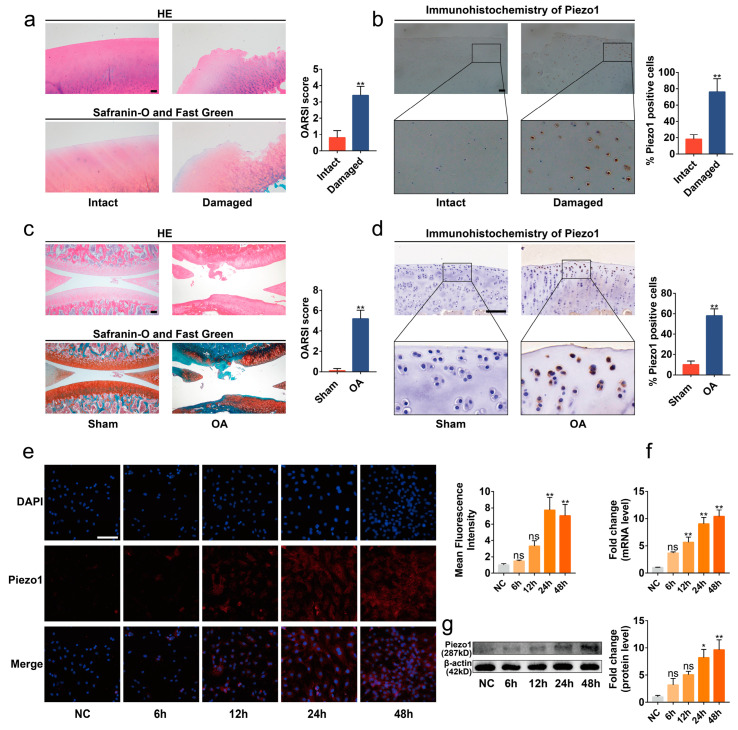
Piezo1 was up-regulated in the articular cartilage of OA patients and rats. (**a**,**c**) Representative images of HE, Safranin-O/Fast Green staining of intact and damaged areas in OA patients’ articular, Sham, and OA rat cartilage, and OARSI scores (*n* = 5). (**b**,**d**) Immunohistochemistry staining of intact and damaged areas in OA patients’ articular, Sham, and OA rat cartilage, and the percentage of Piezo1 positive cells (*n* = 5). (**e**) Immunofluorescence staining and quantified results of Piezo1 in chondrocytes exposed to mechanical strain for 0 h, 6 h, 12 h, 24 h, and 48 h (*n* = 3). (**f**,**g**) RT-qPCR and Western blot analysis of Piezo1 in chondrocytes exposed to mechanical strain for 0 h, 6 h, 12 h, 24 h, and 48 h. * *p* < 0.05; ** *p* < 0.01; ns, no significant differences. Mann–Whitney U test for (**a**,**c**), Student’s *t*-test for (**b**,**d**), one-way ANOVA with Bonferroni’s test for (**e**–**g**); scale bar: 100 μm.

To determine the expression of Piezo1 in OA cartilage, we collected the articular cartilage tissues of OA patients undergoing total knee arthroplasty, and the expression of Piezo1 was evaluated in the damaged or intact area. Significant cartilage degeneration was observed in the damaged area of the articular cartilage compared with the intact area via hematoxylin and eosin (HE) and Safranin-O/Fast Green stains (Figure 1a, *p* < 0.01). Immunohistochemistry analysis showed that the level of Piezo1 protein in the damaged area was elevated in contrast with the intact area (Figure 1b, *p* < 0.01). Similarly, we found that the Piezo1 was notably up-regulated in the articular cartilage of the ACLT surgery-induced OA rats (Figure 1c,d, *p* < 0.01).

Next, the chondrocytes were isolated from immature rats to explore the expression of Piezo1 in an in vitro OA model. Excessive mechanical strain is the key pathogenic mechanism in the development of OA [3]. Accordingly, chondrocytes were cultured in the Flexcell Tension System to simulate the excessive mechanical strain suffered in the progression of OA (Figure S1a). The results of immunofluorescence, quantitative real-time PCR (RT-qPCR), and Western blots showed that the mRNA and protein levels of Piezo1 were increased in the chondrocytes exposed to cyclic mechanical strain (1 Hz, 20%) in a time-dependent manner (Figure 1e–g, *p* < 0.01). Thus, probably due to the mechanical strain, the level of Piezo1 was up-regulated in the articular cartilage of OA, especially in the damaged area.

### 2.2. Excessive Mechanical Strain Induced the Apoptosis and Anabolic/Catabolic Imbalance in Chondrocytes through Piezo1

We next determined the function of Piezo1 in the signal transduction of mechanical strain. Bright-field images showed that the morphology of chondrocytes changed from polygonal to the spindle and apoptotic bodies presented after exposure to the 12 h mechanical strain (Figure S1b,c). Moreover, mechanical strain led to apoptosis and the anabolic and catabolic imbalance in chondrocytes in a time-dependent manner (Figures S2 and S3a,b, *p* < 0.05). Given the impact of mechanical strain on chondrocytes reaching the threshold at 24 h, the 24 h mechanical strain was used in the later experiment.

**Figure 2:**
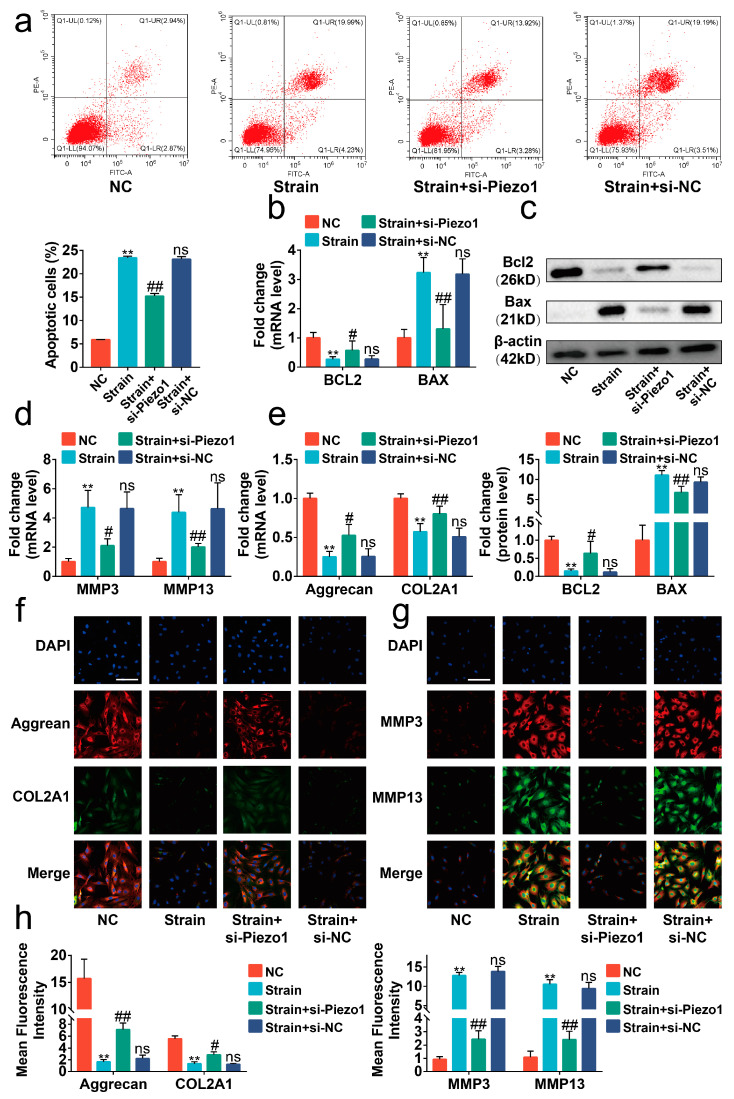
Mechanical strain induced the apoptosis and anabolic/catabolic imbalance in chondrocytes through Piezo1. (**a**) Flow cytometry analysis of chondrocyte stained with Annexin V-FITC and PI (*n* = 3). (**b**) The mRNA levels of BAX and BCL2 in chondrocytes were determined by RT-qPCR (*n* = 3). (**c**) Western blot analysis of BAX and BCL2 in chondrocytes (*n* = 3). (**d**,**e**) The mRNA level of MMP3, MMP13, Aggrecan, and COL2A1 in chondrocytes quantified by RT-qPCR (*n* = 3). (**f**,**g**) Immunofluorescence staining of MMP3, MMP13, Aggrecan, and COL2A1 in chondrocytes (*n* = 3). (**h**) Quantified results of immunofluorescence. ** *p* < 0.01 vs. NC; # *p* < 0.05 vs. strain; ## *p* < 0.01 vs. strain; ns, no significant differences. One-way ANOVA with Bonferroni’s test for (**a**–**e**,**h**), Kruskal–Wallis test with Dunn’s multiple comparisons test for Aggrecan in (**d**). Scale bar: 100 μm.

Piezo1 is the critical protein sensing mechanical signaling on the surface of chondrocytes [20]. Thus, si-Piezo1 was administrated on chondrocytes to determine the role of Piezo1 in the transduction of mechanical signaling. The results of Rt-qPCR indicated that si-Piezo1 could reduce the mRNA level of Piezo1 (Figure S4, *p* < 0.01). We found that si-Piezo1 reversed the increasing proportions of apoptotic cells and altered expression in apoptosis-associated molecules such as BCL2-associated X protein (BAX) and B cell leukemia/lymphoma 2 (BCL2) evoked by mechanical strain (Figure 2a–c, *p* < 0.05). In addition, si-Piezo1 rebalanced the cartilage matrix anabolic and catabolic metabolism damaged by mechanical strain (Figure 2d–h, *p* < 0.05).

**Figure 3:**
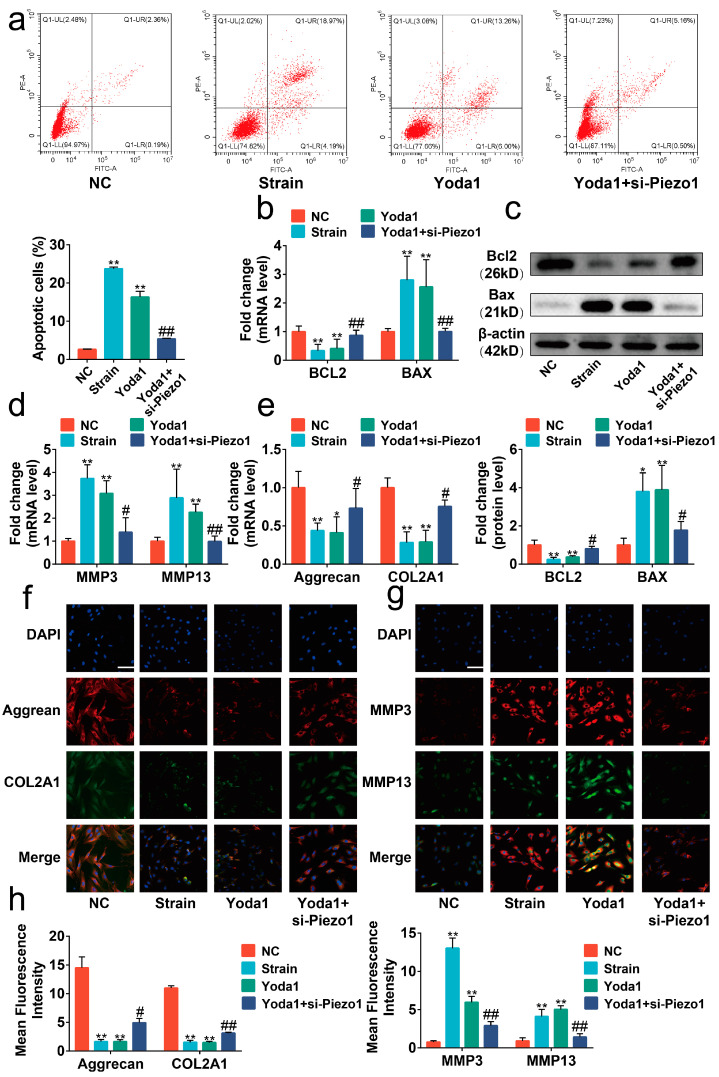
Mechanical strain induced the apoptosis and anabolic/catabolic imbalance in chondrocytes through Piezo1. (**a**) Flow cytometry analysis of chondrocyte stained with Annexin V-FITC and PI (*n* = 3). (**b**) RT-qPCR analysis of BAX and BCL2 in chondrocytes (*n* = 3). (**c**) Protein level of BAX and BCL2 determined by Western blots in chondrocytes (*n* = 3). (**d**,**e**) RT-qPCR analysis of MMP3, MMP13, Aggrecan, and COL2A1 in chondrocytes (*n* = 3). (**f**,**g**) Representative immunofluorescence images of MMP3, MMP13, Aggrecan, and COL2A1 in chondrocytes (*n* = 3). (**h**) Quantified results of immunofluorescence. * *p* < 0.05 vs. NC; ** *p* < 0.01 vs. NC; # *p* < 0.05 vs. Yoda1; ## *p* < 0.01 vs. Yoda1. One-way ANOVA with Bonferroni’s test for (**a**–**e**,**h**), Kruskal–Wallis test with Dunn’s multiple comparisons test for COL2A1 in (**d**) and MMP3 in (**e**). Scale bar: 100 μm.

To further confirm the role of Piezo1 on mechanical signaling conduction, we treated chondrocytes with Yoda1, a synthetic chemical compound that contributes to activating Piezo1 [21]. We found that the effects of Yoda1 treatment were similar to mechanical strain, presenting high proportions of apoptotic cells, altered expression of BAX, BCL2, matrix metallopeptidase 3 (MMP3), matrix metallopeptidase 13 (MMP13), collagen type II alpha 1 chain (COL2A1), and Aggrecan (Figure 3a–h, *p* < 0.05). Additionally, si-Piezo1 could mitigate its impact on apoptosis or anabolic/catabolic imbalance. All the data indicated that mechanical strain induced apoptosis and the anabolic and catabolic imbalance in chondrocytes through up-regulating and activating Piezo1.

### 2.3. Piezo1 Activated CaN/NFAT1 Signaling Axis under Mechanical Strain

**Figure 4:**
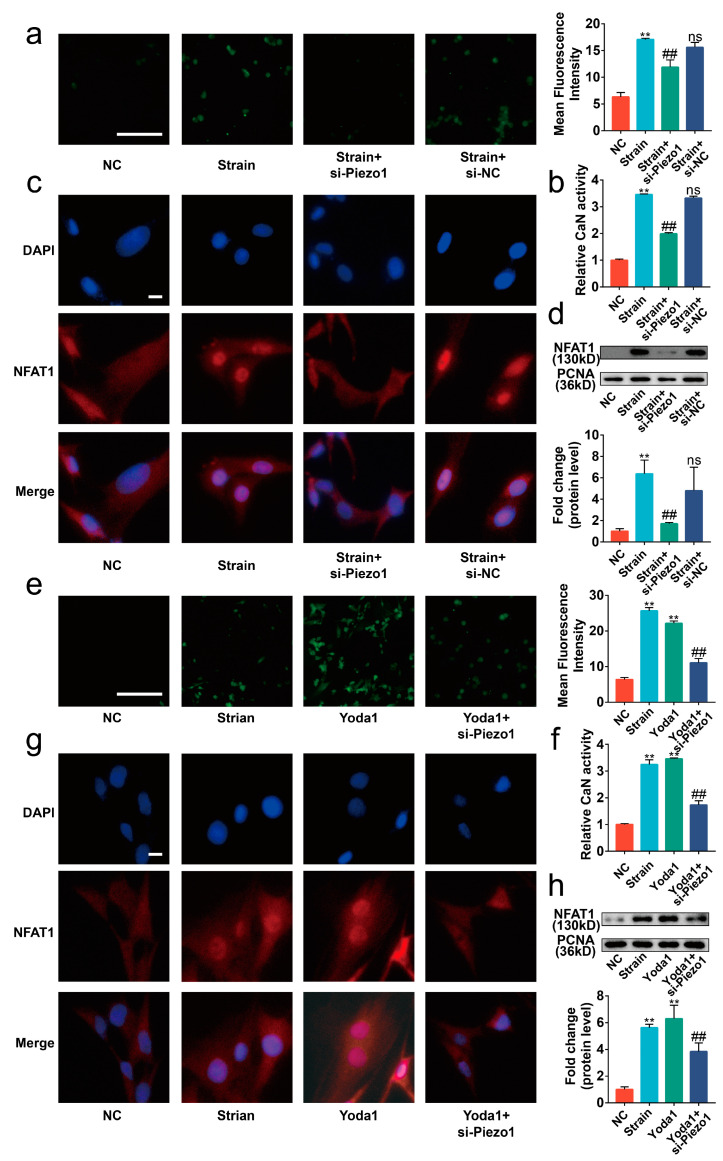
Piezo1 activated the CaN/NFAT1 signaling axis under mechanical strain. (**a**,**e**) Representative image of the Flou-4 AM stain (*n* = 3), scale bar: 100 μm. (**b**,**f**) The activity of CaN is determined by the CaN Activity Kit (*n* = 3). (**c**,**g**) Representative immunofluorescence image of NFAT1 nuclear translocation in each group (*n* = 3), scale bar: 10 μm. (**d**,**h**) Western blot analysis of NFAT1 in chondrocyte nucleoprotein (*n* = 3). ** *p* < 0.01 vs. NC; ## *p* < 0.01 vs. strain or Yoda1; ns, no significant differences. One-way ANOVA with Bonferroni’s test for (**a**,**b**,**d**–**f**,**h**).

Upon Piezo1 activation, calcium increases in the cytoplasm via calcium influx [20]. Thus, we focused on the CaN/ NFAT signaling axis, a major downstream pathway of calcium signaling, to elucidate the mechanism of Piezo1-induced apoptosis and anabolic/catabolic imbalance in chondrocytes [22]. Our data showed that si-Piezo1 suppressed the calcium influx and the activity of CaN induced by mechanical strain (Figure 4a,b, *p* < 0.01). Immunofluorescence and Western blot results indicated that the nuclear translocation of NFAT1 promoted by mechanical strain was reversed by si-Piezo1 (Figure 4c,d, *p* < 0.01). Next, we administrated Yoda1 on chondrocytes to further explore the impact of Piezo1 on the CaN/NFAT1 signaling axis. Similar to mechanical strain, Yoda1 had positive effects on calcium influx, CaN activity, and the nuclear translocation of NFAT1, which were reversed by si-Piezo1 (Figure 4e–g, *p* < 0.01). Collectively, the results indicate that mechanical strain activated the CaN/NFAT1 signaling axis by up-regulating the expression and activating Piezo1.

### 2.4. Blocking the Piezo1/CaN/NFAT1 Signaling Axis Inhibited the Deleterious Effects of Mechanical Strain

**Figure 5:**
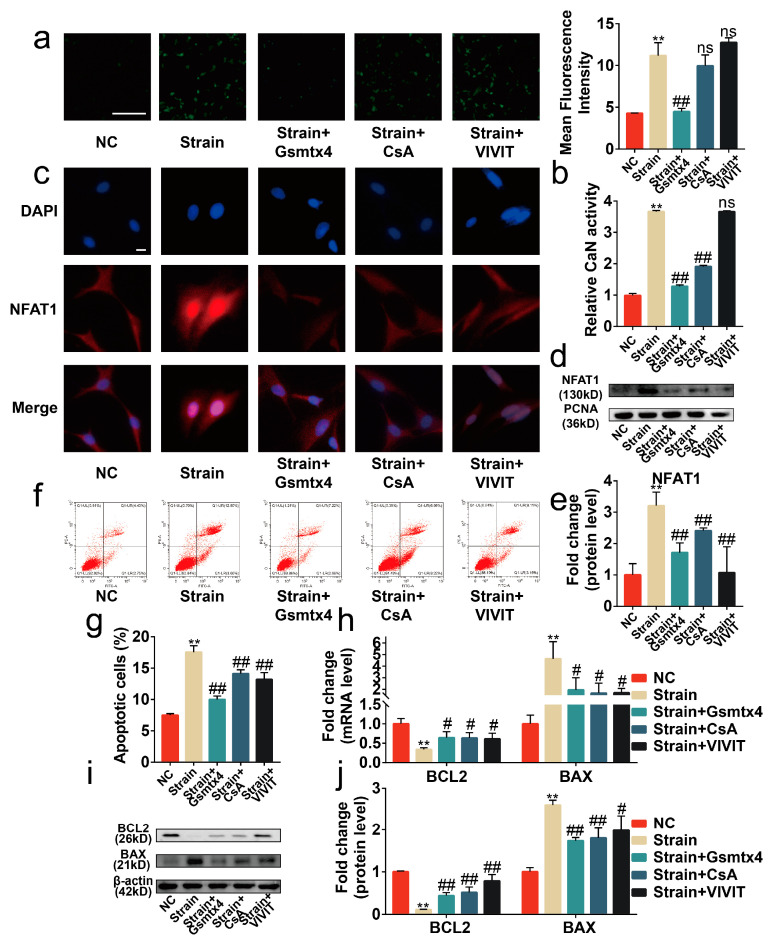
Blocking the signal transduction of the CaN/NFAT1 signaling axis protected chondrocytes from apoptosis. (**a**) Representative image of the Flou-4AM stain (*n* = 3), scale bar: 100 μm. (**b**) The activity of CaN is determined by the CaN Activity Kit (*n* = 3). (**c**) Representative immunofluorescence image of NFAT1 nuclear translocation (*n* = 3), scale bar: 10 μm. (**d**) Western blot analysis of NFAT1 in chondrocyte nucleoprotein (*n* = 3). (**e**) Quantified results of (**d**). (**f**,**g**) Flow cytometry analysis of chondrocyte stained with Annexin V-FITC and PI (*n* = 3). (**h**) mRNA levels of BAX and BCL2 in chondrocytes determined by RT-qPCR (*n* = 3). (**i**,**j**) Western blot analysis and quantified results of BAX and BCL2 (*n* = 3). ** *p* < 0.01 vs. NC; # *p* < 0.05 vs. strain; ## *p* < 0.01 vs. strain; ns, no significant differences. One-way ANOVA with Bonferroni’s test for (**a**,**b**,**e**,**g**,**j**), Kruskal–Wallis test with Dunn’s multiple comparisons test for (**h**). Scale bar: 100 μm.

**Figure 6:**
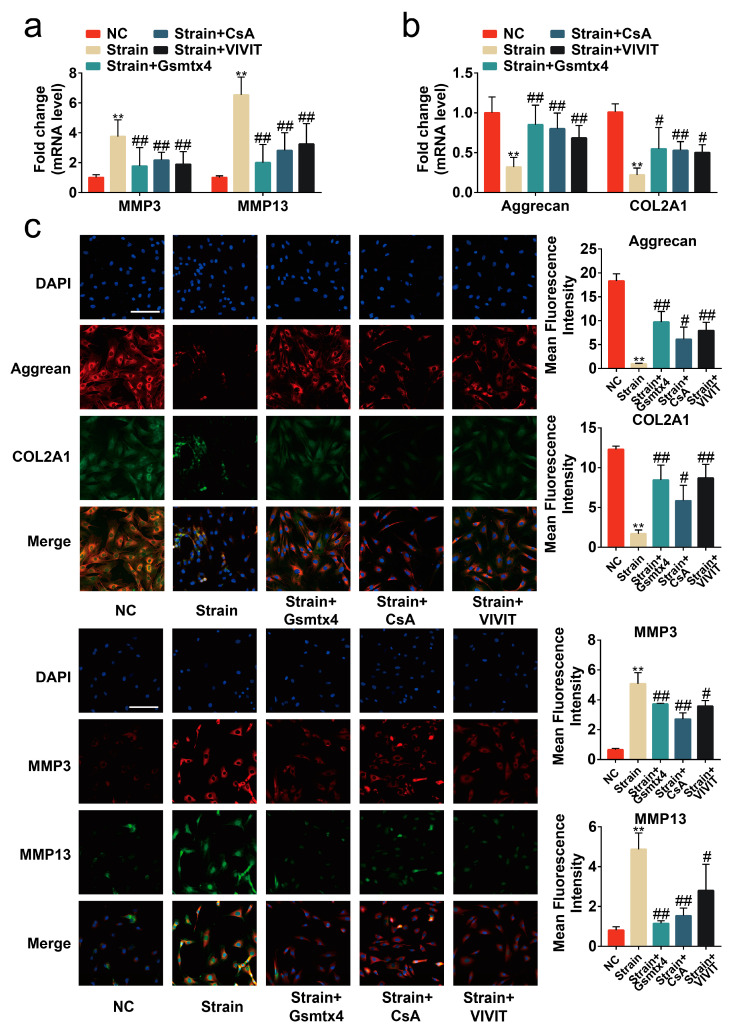
Blocking the signal transduction of the CaN/NFAT1 signaling axis protected chondrocytes from anabolic/catabolic imbalance. (**a**,**b**) mRNA levels of Aggrecan, COL2A1, MMP3, and MMP13 in chondrocytes determined by RT-qPCR (*n* = 3). (**c**) Immunofluorescence staining and quantified results of MMP3, MMP13, Aggrecan, and COL2A1 in chondrocytes (*n* = 3). ** *p* < 0.01 vs. NC; # *p* < 0.05 vs. strain; ## *p* < 0.01 vs. strain;. Scale bar: 100μm. One-way ANOVA with Bonferroni’s test for (**a**–**c**), Kruskal–Wallis test with Dunn’s multiple comparisons test for COL2A1 in (**b**).

To assess the necessity of the Piezo1/CaN/NFAT1 signaling axis on the mechanical signaling transduction, Gsmtx4, the CaN inhibitor CsA, and the NFAT inhibitor VIVIT peptide were used to specifically block the signal transduction of the CaN/NFAT1 signaling axis [15,23,24]. Our results revealed that the intracellular calcium concentration was reduced by Gsmtx4 but was almost unaffected by CsA and the VIVIT peptide (Figure 5a, *p* < 0.01). Concurrently, both CsA and Gsmtx4 could inactivate CaN in mechanical-strain-treated chondrocytes (Figure 5b, *p* < 0.01). The results of immunofluorescence and Western blot showed that Gsmtx4, CsA, and VIVIT inhibited the nuclear translocation of NFAT1 (Figure 5c–e, *p* < 0.01). Finally, we found that Gsmtx4, CsA, and VIVIT could protect chondrocytes from apoptosis and the anabolic and catabolic imbalance (Figure 5f–j and Figure 6a–c, *p* < 0.01). Thus, the Piezo1/CaN/NFAT1 signaling axis was necessary for the mechanical signaling transduction and blocking the Piezo1/CaN/NFAT1 signaling axis could protect chondrocytes from apoptosis and anabolic/catabolic imbalance under mechanical strain.

### 2.5. Intra-Articular Injections of Gsmtx4 Ameliorated OA in Rats

**Figure 7:**
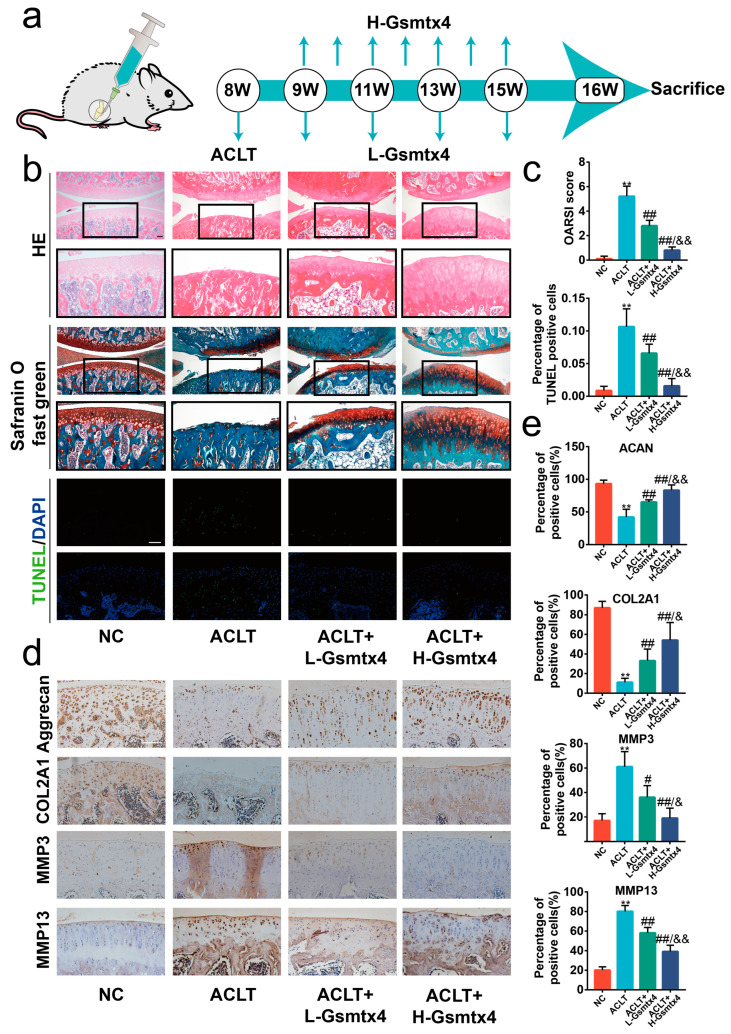
Intra-articular injections of Gsmtx4 ameliorated OA in rats. (**a**) Schematic illustration of Gsmtx4 injection in the ACLT-induced OA rat model. (**b**) HE, Safranin-O/Fast Green, and TUNEL stains of articular cartilage (*n* = 5). (**c**) The OARIS scores and the percentage of TUNEL positive cells of the cartilage (*n* = 5). (**d**) Immunohistochemistry staining of Aggrecan, COL2A1, MMP3, and MMP13 in articular cartilage, and (**e**) percentage of positive cells (*n* = 5). ** *p* < 0.01 vs. Sham; # *p* < 0.05 vs. ACLT; ## *p* < 0.01 vs. ACLT; & *p* < 0.05 vs. L-Gsmtx4; && *p* < 0.05 vs. L-Gsmtx4. One-way ANOVA with Bonferroni’s test for (**c**,**e**), Kruskal–Wallis test with Dunn’s multiple comparisons test for OARSI score in (**c**). Scale bar: 100 μm.

ACLT surgery, one of the most traditional ways to harvest the OA model, leads to joint instability and altered joint mechanics [25]. Thus, we sought to investigate the effects of blocking the Piezo1/CaN/NFAT1 signaling axis by intra-articular injections of Gsmtx4 on ACLT-induced OA rats. Considering the fast clearance in synovial fluid, we administered the injections at two different frequencies (once a week in the H-Gsmtx4 group and once every two weeks in the L-Gsmtx4 group) (Figure 7a) [26]. HE and Safranin O/Fast Green staining showed erosion on the articular cartilage surfaces and proteoglycan loss in the ACLT group after eight weeks following surgery. Compared with the ACLT group, the ACLT+L-Gsmtx4 group and the ACLT+H-Gsmtx4 group had mild destruction of articular cartilage, especially in the latter (Figure 7b). Moreover, Osteoarthritis Research Society International (OARSI) scores were notably lower in the group with Gsmtx4 treatment in contrast with the ACLT group, and ACLT+H-Gsmtx4 groups were lower than the ACLT+L-Gsmtx4 group. The articular cartilage terminal deoxynucleotidyl transferase dUTP nick end labeling (TUNEL) staining showed that Gsmtx4 reduced the proportion of apoptotic chondrocytes, which was dose-dependent (Figure 7c, *p* < 0.01). Additionally, the results of immunohistochemical staining revealed that the Gsmtx4 injection reduced the expression of MMP3 and MMP13 and increased the expression of COL2A1 and Aggrecan in the ACLT-induced OA model (Figure 7d, *p* < 0.01). Among them, intra-articular injection of Gsmtx4 with high frequency exhibited better efficacy. In summary, these results demonstrated that Gsmtx4 could ameliorate OA in rats by suppressing apoptosis and the anabolic and catabolic imbalance.

## 3. Discussion

The etiology of OA is multifactorial, such as age, obesity, high level of joint strain, and injury [27]. Among them, excessive mechanical strain is regarded as the essence of a series of risk factors in the progression of OA [28]. Growing evidence suggests that excessive mechanical strain contributed to pathological changes in chondrocytes, including senescence, apoptosis, and oxidative stress [10,29]. Therefore, it is crucial to elucidate the underlying mechanism by which excessive mechanical strain aggravated OA.

The role of Piezo1, a crucial mechanosensitive ion channel, in the development of OA has been controversial in previous studies. Lee et al. observed an elevated level of Piezo1 in the interleukin-1β-induced OA chondrocyte model which sensitized mechanotransduction in the articular cartilage [30]. However, Ryo et al. believed that the expression of Piezo was unaffected in iodoacetate-induced OA rats [31]. Nevertheless, Piezo1-mediated apoptosis induced by mechanical strain has been confirmed in multiple cell types, such as macrophages and intervertebral disc cells [29,32]. In the present study, we demonstrated that Piezo1 was significantly up-regulated in the articular cartilage of OA patients and ACLT-induced OA rats. Meanwhile, we observed the increased Piezo1 expression in IL-1β or mechanical-strain-treated chondrocytes, which corroborated that the rising level of Piezo1 was attributable to mechanical strain and inflammatory stimuli. Furthermore, we determined that the elevated expression and activity of Piezo1 contributed to the transduction of abnormal mechanical signaling and aggravated OA. Thus, our finding demonstrated that Piezo1 contributed to OA development.

Piezo1 acts through leading calcium influx, and the rising calcium concentration has been reported in various cells under activated Piezo1 [33,34]. Calcium ions are the major second messengers involved in many physiologies and pathophysiologies such as proliferation, apoptosis, and autophagy [35]. Elevated calcium ion was observed in the OA cartilage and was closely associated with apoptosis and senescence [36]. Our results confirmed the increased calcium ion concentration under mechanical strain. Therefore, mechanical signaling conduction in the cartilage was mediated by calcium ions. Although most research focused on the transient calcium influx under activated Piezo1, increased resting calcium ion content was also reflected in their data [37]. The same phenomenon is observed in this study. The activity of CaN/NFAT is dependent on the repetitive or prolonged increase in intracellular calcium ions [38]. The enhanced transcription of inflammatory cytokines such as interleukin-17 and tumor necrosis factor-α was the ultimate effect of CaN/NFAT1 activation [39]. In OA, increased activity of CaN was found in chondrocytes and contributed to the secretion of inflammatory cytokines and anabolic and catabolic imbalances of the cartilage matrix [16]. Moreover, NFAT1 is considered an important transcription factor in maintaining cartilage development and cartilage anabolism and catabolism [40]. Adult mice lacking the transcription factor NFAT1 exhibit spontaneous OA [41]. The extrusive activation of NFAT1 in inflammatory states promotes the inflammatory phenotype [42]. Therefore, CaN/NFAT1 may be the downstream effector proteins of Piezo1 activation and mediate the chondrocyte pathological phenotype. In this study, we found that CaN/NFAT1 was activated by mechanical strain and dependent on the activation of Piezo1 and intracellular calcium concentration. Inhibitors of CaN or NFAT1 could block the apoptosis and anabolic and catabolic imbalance in chondrocytes under mechanical strain or activated Piezo1. Our results confirmed that CaN/NFAT1 is a critical downstream signal of Piezo1.

The toxin peptide Gsmtx4 is a Piezo1 inhibitor, and a previous study reported that Gsmtx4 could protect chondrocytes from cell death following injury in cartilage explants [23]. In the present study, we determined that Gsmtx4 inhibited chondrocyte apoptosis and maintained metabolic homeostasis, and we characterized the blocking effects of Gsmtx4 in the CaN/NFAT1 signaling axis in more detail. He et al. suggested that the intra-articular injection of Gsmtx4 reduced the primary mechanical allodynia by inhibiting nerve conduction in OA mice [43]. However, the effect of Gsmtx4 in alleviating OA progression remains unclear. In our results, Gsmtx4 preserved cartilage integrity, reduced the percentage of apoptosis chondrocytes, and maintained metabolic homeostasis of cartilage, especially in the high-frequency intra-articular injection group. Thus, Gsmtx4 may be the novel therapeutic strategy for OA patients in relieving symptoms or slowing progression.

However, there are several limitations to this study. First, we focused on the baseline concentrations of calcium, but the transient change may need to be described. Additionally, highly efficient means of Gsmtx4 delivery need to be developed to avoid the infection risks of high-frequency injections.

## 4. Materials and Methods

### 4.1. Ethics Statement

The collection and experiments of human cartilage samples were given informed consent by patients and approved by the Ethical Committee of Renmin Hospital of Wuhan University (Ethic code: WDRY2022-K223). All experiments involving animals were consistent with the National Research Council’s Guide for the Care and Use of Laboratory Animals and were approved by the Laboratory Animal Welfare and Ethics Committee of the Renmin Hospital of Wuhan University (Approval No: 20220103A).

### 4.2. Human Samples

The cartilage tissues were collected from the femoral medial and lateral condyle of five OA patients who underwent total knee arthroplasty at Renmin Hospital of Wuhan University. Collected cartilage tissues were fixed in 4% paraformaldehyde for 24 h and decalcified with 0.5 M ethylene diamine tetraacetic acid (EDTA) until soft. According to the International Cartilage Repair Society (ICRS) grade [44], we selected and isolated one representative cartilage area with ICRS grade I as the intact group and one with ICRS grade IV as the damaged group from each sample. Then, five intact cartilage tissues and five damaged cartilage tissues were cut into 5–10 mm blocks. After embedding in paraffin, cartilage tissues were stored until needed at 4 °C. Patients’ information including gender, age, the surgical site, and ICRS grades is summarized in Table S1.

### 4.3. OA Rat Model

Twenty 8-week-old male Wistar rats were purchased from SiPeiFu Biotechnology Co., Ltd. (Beijing, China). As previously described, OA was induced by ACLT [45]. In brief, rats were anesthetized with 1.5–5% isoflurane inhalation anesthesia. After that, the joint cavity was exposed and the anterior cruciate was cut off. The contralateral capsule was cut as a Sham control. After surgery, OA rats were randomly grouped into four groups with five rats in each group: Sham, ACLT, ACLT + low-frequency injection of Gsmtx4 (HY-P1410, MCE, Shanghai, China, L-Gsmtx4), ACLT + high-frequency injection of Gsmtx4 (H-Gsmtx4). An amount of 100 μL of 40 μM Gsmtx4 was injected intra-articularly at 7, 14, 21, 28, 35, 42, and 49 days following surgery for the ACLT + H-Gsmtx4 group, and at 7, 21, 35, and 49 days following surgery for the ACLT + L-Gsmtx4 group. At eight weeks after surgery, right knee joints of twenty rats were collected. After fixing with 4% paraformaldehyde for one day and decalcifying with 0.5 M EDTA for 7–30 days, the rat cartilage samples were embedded in paraffin and stored at 4 °C for further studies.

### 4.4. Histology and Immunohistochemistry

Serial sagittal sections of embedded human and rat samples were sectioned in 6 μm and stained with HE and Safranin-O/Fast Green. OARSI scoring was used to degrade the severity of OA as reported previously [46]. For immunohistochemistry experiments, sections were incubated overnight at 4 °C with anti-MMP3 (1:200, A1202; Abclonal, Wuhan, China), anti-MMP13 (1:200, GB11247; Servicebio, Wuhan, China), anti-Aggrecan (1:400, TD7561; Abmart, Shanghai, China), and anti-COL2A1 (1:400, TA0135; Abmart, Shanghai, China). Sections were visualized using an HRP detection system and the rate of positive cells was quantified by two blinded pathologists.

### 4.5. Cell Culture and Intervention

Primary chondrocytes were extracted from the cartilage of 6-day-old rats as conducted previously [47]. In a nutshell, cartilage tissue was cut into pieces and digested for 8 h with 0.2% collagenase II. Isolated chondrocytes were cultured in DMEM/F12 medium supplemented with 0.5% penicillin/streptomycin and 10% fetal bovine serum. For mechanical strain intervention, chondrocytes were seeded on 6-well Flexcell culture plates and imparted at 20% elongation, 0.1 Hz frequency through the Flexcell Tension System (FX4000T; FlexCell International Corporation, Burlington, NC, USA) [48]. For pharmacological interventions, Gsmtx4 (40 μM) [23], NFAT inhibitor-1 VIVIT (10 μM, HY-P1026, MCE, Shanghai, China) [24], Cyclosporin A (1 μM, HY-B0579, MCE, Shanghai, China) [15], and Yoda1 (5 μM, HY-18723, MCE, Shanghai, China) [47] were added to the medium for 24 h.

### 4.6. Quantitative Transcript Analyses

Total RNA was extracted by the RNA extraction kit (R0027, Beyotime, Shanghai, China). Furthermore, 1μg of RNA was reverse transcribed into cDNA using the cDNA synthesis kit (G3330-100, Servicebio, Wuhan, China). qRT-PCR was performed with the SYBR Green PCR Master Mix (G3326-01, Servicebio, Wuhan, China) on a Roche LightCycler480^®^ system (Roche, Switzerland). Primers used in this study are listed in Table S2.

### 4.7. Immunofluorescence

Chondrocytes grown on the 6-well Flexcell culture plates were transferred to glass coverslips. After adhesion, chondrocytes were rinsed with PBS and fixed with 4% paraformaldehyde at room temperature for 15 min, then rinsed with PBS. Then, 1% Triton X-100 and 5% normal goat serum were added for permeabilizing for 15 min and binding nonspecific protein for 1 h, respectively. After that, chondrocytes were incubated with anti-Piezo1 (1:200, DF12083, Affinity, Liyang, China), anti-NFAT1 (1:200, A3107, Abclonal, Wuhan, China), anti-MMP3 (1:100, A1202; Abclonal, Wuhan, China), anti-MMP13 (1:200, GB11247; Servicebio, Wuhan, China), anti-COL2A1 (1:200, TA0135; Abmart, Shanghai, China), and anti-Aggrecan (1:200, TD7561; Abmart, Shanghai, China) antibodies overnight at 4 °C. After rinsing with PBST, chondrocytes were incubated with anti-mouse or anti-rabbit conjugated with CY3 and FITC for 1 h at room temperature. Next, chondrocytes were rinsed with PBST and stained with DAPI. Images were captured randomly on an Olympus BX53 fluorescence microscope. ImageJ software (version: 1.8.0) was used to quantify the mean immunofluorescent intensity.

### 4.8. Western Blot

The total protein or nuclear protein of chondrocytes was extracted using a protein extraction kit (WLA019, Wanleibio, Shenyang, China) and a nuclear protein extraction kit (P0028, Beyotime, Shanghai, China). The protein concentration was assessed by BCA kit (G2026, Servicebio, Wuhan, China). Forty-gram proteins per sample were separated by 10% SDS-PAGE and transferred to polyvinylidene difluoride membranes. The membranes were blocked for 2 h with 5% skim milk, and incubation with anti-BAX (1:2000, 50599, Proteintech, Wuhan, China), anti-BCL2 (1:500, A0208, Abclonal, Wuhan, China), anti-β-ACTIN (1:2000, GB11001, Servicebio, Wuhan, China), anti-NFAT1 (1:1000, A3107, Abclonal), and anti-PCNA (1:1000, GB11010, Servicebio, Wuhan, China) at 4 °C was performed overnight. After incubation with HRP-conjugated secondary antibodies, target bands were visualized using the ChemiDoc Touch (Bio-Rad, CA, USA).

### 4.9. Apoptosis Assay

In vitro, chondrocyte apoptosis was assessed using Annexin V-FITC/PI Apoptosis Detection Kit (40302ES20, Yeason, Shanghai, China) according to the manufacturer’s protocol. In vivo, apoptosis degrades were tested via In Situ Cell Death Detection Kit (11684817910, Roche, Basel, Switzerland), and TUNEL-positive chondrocytes were counted on five different fields.

### 4.10. RNA Intervention

The target sequence for si-Piezo1 (siB140821184040-1-5, Ribobio, Guangzhou, China) was as follows: CGGCCAACATAAAGAACAT. Chondrocytes were transfected with si-Piezo1 or si-NC using Optimem medium and Lipofectamine 2000 (#11668019, Invitrogen, NY, USA). After six hours, the Optimem medium was replaced by DMEM/F12 medium, and the effect of RNA intervention was evaluated by RT-qPCR.

### 4.11. Calcium Ions

Flou-4 AM (S1060, Beyotime, Shanghai, China) loading was performed to measure intracellular calcium content. Following the manufacturer’s protocol, Flou-4 AM was diluted to 1 μM with PBS. After washing with HBSS, chondrocytes were incubated with Flou-4 AM for 30 min. Images were obtained through an Olympus IX50 inverted microscope and quantified by ImageJ software (version: 1.8.0).

### 4.12. Calcineurin Activity

The calcineurin activity was evaluated by a calcineurin assay kit (A068-1-1, NanJing Jiancheng Bio, NanJing, China) following the manufacturer’s protocol. The absorbance was measured by a microplate reader (EnVision, PerkinElmer, Waltham, CA, USA).

### 4.13. Statistical Analysis

Statistical analyses and data visualization were carried out in the Prism software (version: 7.0.0). All graphical data are displayed as the mean  ±  SD. Shapiro–Wilk normality test was used to perform the normality test. For the data with normal distribution, Student’s *t*-test (unpaired, two groups, equal variances), Welch’s *t*-test (unpaired, two groups, unequal variances), and one-way analysis of variance (ANOVA) followed by Bonferroni’s test (multiple groups) were administrated. For the data with non-normal distribution, we performed the Mann–Whitney U test (two groups) and Kruskal–Wallis H test, followed by Dunn’s test (multiple groups). All statistical tests were two-tailed, and a *p*-value < 0.05 was considered significant.

## 5. Conclusions

**Figure 8:**
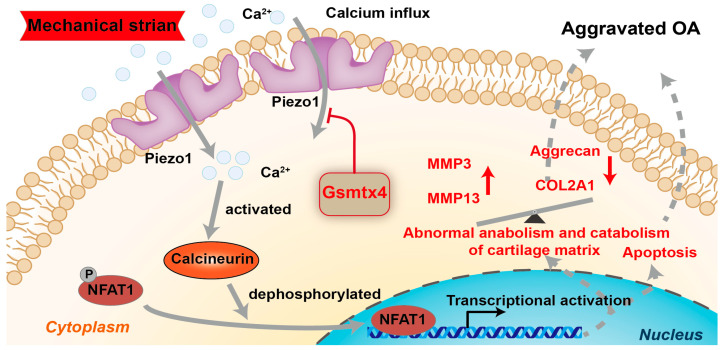
Schematic diagram representing the molecular mechanism by which Gsmtx4 alleviated OA under excessive mechanical strain.

In conclusion, we demonstrated that Piezo1 was up-regulated and activated under mechanical strain, and caused apoptosis and anabolic/catabolic imbalance in chondrocytes through the CaN/NFAT1 signaling axis (Figure 8). The Piezo1 inhibitor Gsmtx4 alleviated OA both in vivo and in vitro. The results support that Piezo1/CaN/NFAT1 may be the promising therapeutic target for OA.

## Data Availability

The data presented in this study are available in the article or Supplementary Material.

## Supplementary Materials

The following supporting information can be downloaded at: https://www.mdpi.com/article/10.3390/ijms24044022/s1.
