# Supplementary material for: Gsmtx4 Alleviated Osteoarthritis through Piezo1/Calcineurin/NFAT1 Signaling Axis under Excessive Mechanical Strain
Source: Int J Mol Sci. 2023 Feb 16;24(4):4022. doi: 10.3390/ijms24044022 (PMC9961447; doi:10.3390/ijms24044022)
Supplement: Supplementary file 1 [file ijms-24-04022-s001.zip › ijms-2201084-supplementary.pdf]

Supplementary Materials

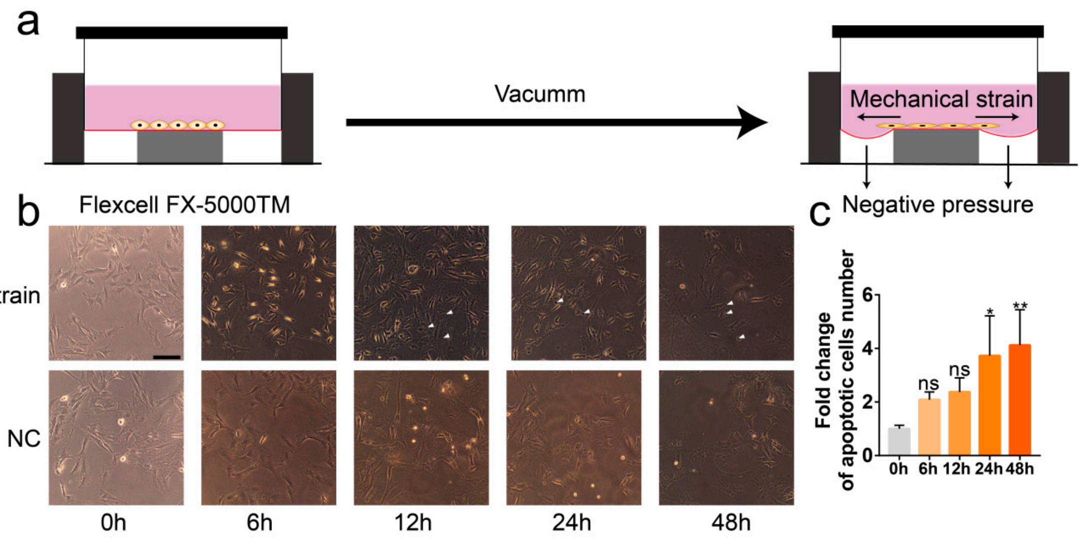

Figure S1. Morphological changes of chondrocytes under mechanical strain. **(a)** A schematic diagram of the mechanical strain treatment on chondrocytes. **(b)** The morphological characteristics of chondrocytes undergoing mechanical strain. **(c)** Quantified analysis of apoptotic chondrocytes (*n*=3). *, $p < 0.05$ vs NC; **, $p < 0.01$ vs NC; ns, no significant differences. one-way ANOVA with Bonferroni's test for **(c)**. Scale bar: 100μm.

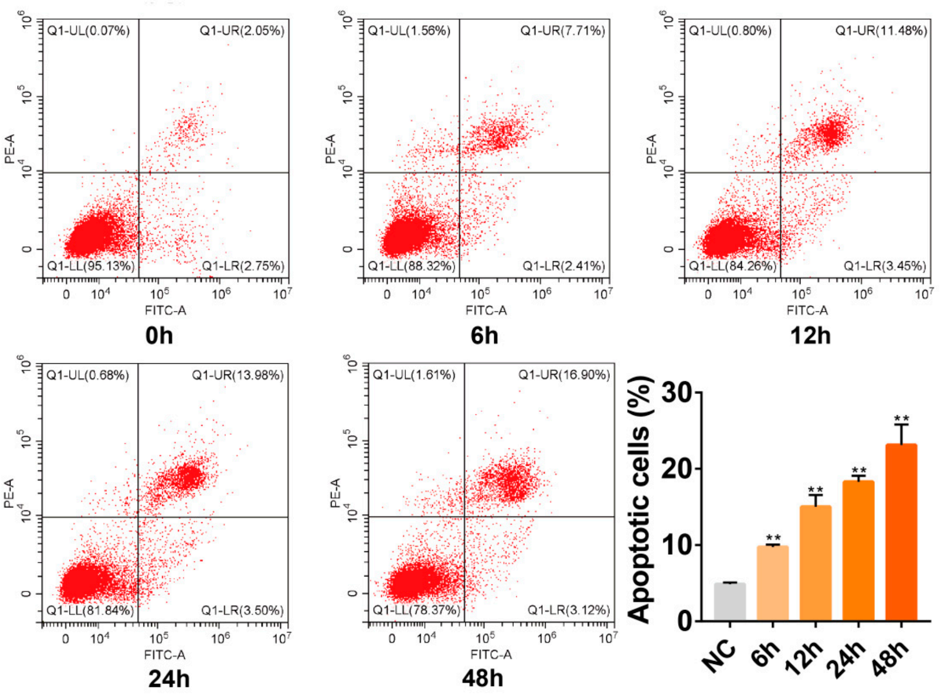

Figure S2. Flow cytometry analysis of chondrocyte stained with Annexin V-FITC and PI (*n*=3). **, $p < 0.01$ vs NC. one-way ANOVA with Bonferroni's test was performed the statistical analysis.

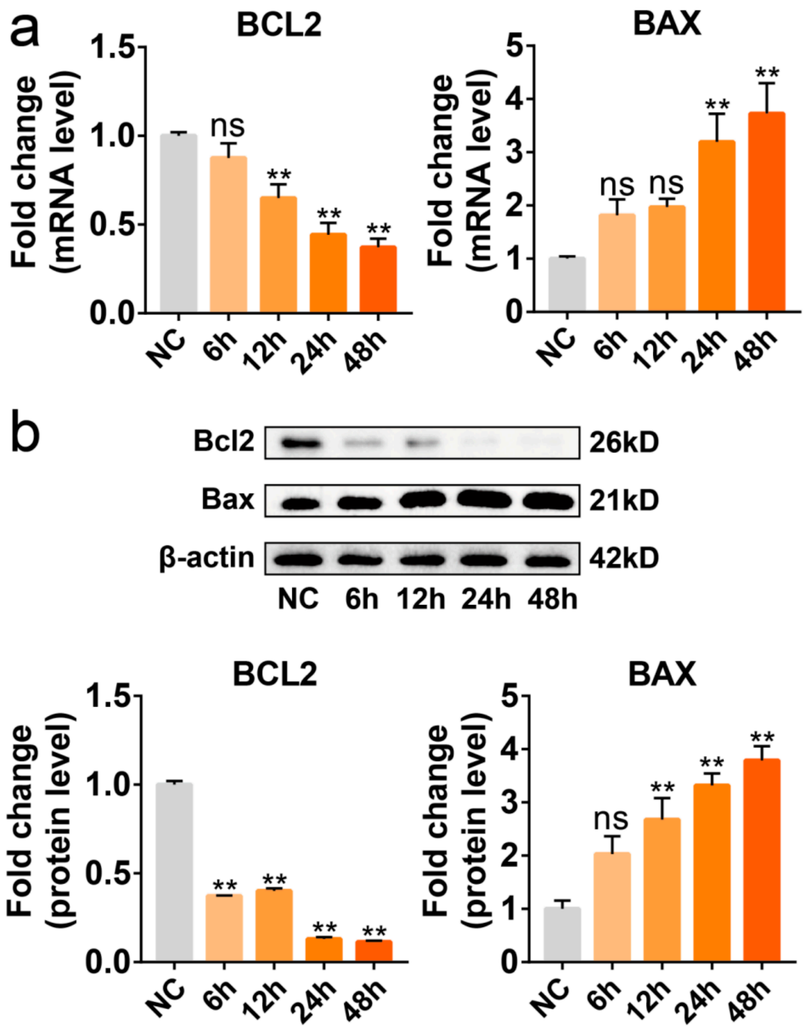

Figure S3. Mechanical strain led to apoptosis and anabolic/catabolic imbalance in chondrocytes in a time-dependent manner. **(a)** RT-qPCR analysis of BAX and BCL2 in chondrocytes exposed to mechanical strain (n=3). **(b)** Western blots analysis of BAX and BCL2 in chondrocytes exposed to mechanical strain (n=3). **, $p < 0.01$ vs NC; ns, no significant differences. one-way ANOVA with Bonferroni's test for **(a, b)**.

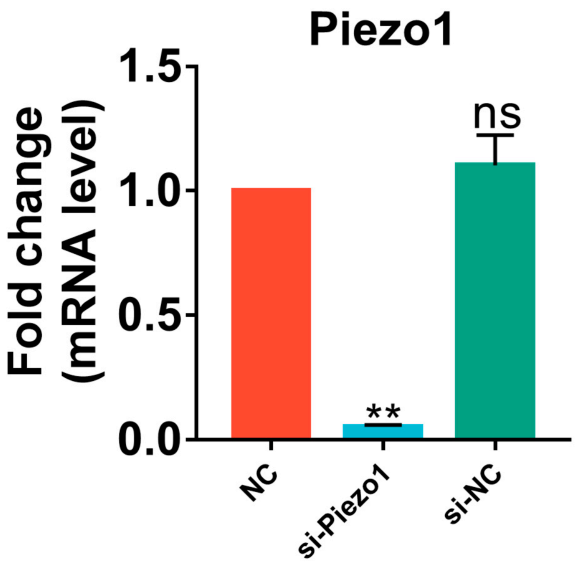

Figure S4. The efficiency of si-Piezo1 investigated by qRT-PCR (*n*=3). **, $p < 0.01$ vs NC; ns, no significant differences. one-way ANOVA with Bonferroni's test for the statistical analysis.

Table S1. Clinical information of human samples

ID	Age	Gender	Surgical site	ICRS
1	70	F	Right knee and Left knee	Intact: I<br>Damaged: IV
2	68	F	Right knee	Intact: I<br>Damaged: IV
3	82	F	Left knee	Intact: I<br>Damaged: IV
4	73	F	Left knee	Intact: I<br>Damaged: IV
5	59	R	Right knee	Intact: I<br>Damaged: IV

Table S2. Primer sequences used in this study

**Gene name**	**F/R**	**Sequences 5'-3'**
β-actin	F	TGCTATGTTGCCCTAGACTTCG
	R	GTTGGCATAGAGGTCTTTACGG
Piezo1	F	AGCAAGCAGGCACAAAGGC
	R	CGCACAAACTTGCCAACGAC

Bax	F	TTTTTGCTACAGGGTTTC
	R	TTGTTGTCCAGTTCATCG
Bcl2	F	GAGAGCGTCAACAGGGAG
	R	GCCAGGAGAAATCAAACA
Mmp3	F	CATGAACTTGGCCACTCCCT
	R	TGGGTACCACGAGGACATCA
Mmp13	F	GCCACCTTCTTCTTGTTGAGTTG
	R	GACTTCTTCAGGATTCCCGCA
Aggrecan	F	AGTGACCCATCTGCTTACCCTG
	R	CTGCATCTATGTCGGAGGTAGTG
Col2a1	F	GTGTCAAGGGTCACAGAGGTTAC
	R	CGCTCTCACCCTTCACACCT
